# TrkB phosphorylation in serum extracellular vesicles correlates with cognitive function enhanced by ergothioneine in humans

**DOI:** 10.1038/s41538-024-00250-5

**Published:** 2024-02-06

**Authors:** Takahiro Ishimoto, Reiya Yamashita, Ruri Matsumoto, Satoshi Matsumoto, Yusuke Matsuo, Shunsuke Nakao, Yusuke Masuo, Makoto Suzuki, Yukio Kato

**Affiliations:** 1 Faculty of Pharmacy, Institute of Medical, Pharmaceutical and Health Sciences, Kanazawa University, Kanazawa, Ishikawa 920-1192 Japan; 2 L·S Corporation Co. Ltd., 3-10-1 Ningyocho-Nihonbashi, Chuo-ku, Tokyo 103-0013 Japan

**Keywords:** Neuroscience, Biomarkers

## Abstract

Oral administration of the food-derived antioxidant amino acid ergothioneine (ERGO) results in its efficient distribution in the brain and enhances cognitive function. However, effect of ERGO deficiency on cognitive impairment and the underlying mechanisms remain unknown. We revealed that cognitive function and hippocampal neurogenesis were lower in mice fed an ERGO-free diet than in those fed the control diet. Furthermore, ERGO supplementation to achieve the control diet ERGO levels reversed these effects and restored ERGO concentrations in the plasma and hippocampus. The ERGO-induced recovery of cognitive function and hippocampal neurogenesis was blocked by inhibiting the neurotrophic factor receptor tropomyosin receptor kinase B (TrkB), with a concomitant reduction in hippocampal phosphorylated TrkB, suggesting the involvement of TrkB in these events in mice. Phosphorylated TrkB was also detected in extracellular vesicles (EVs) derived from serum of volunteers who had been orally administered placebo or ERGO-containing tablets. Importantly, the ratio of serum EV-derived phosphorylated TrkB was significantly higher in the ERGO-treated group than in the placebo-treated group and was positively correlated with both serum ERGO concentrations and several cognitive domain scores from Cognitrax. Altogether, TrkB phosphorylation is involved in ERGO-induced cognitive enhancement in mice, and TrkB phosphorylation levels in serum EVs may quantitatively represent ERGO-induced cognitive enhancement in humans.

## Introduction

Over 50 million people worldwide have dementia, and this number is rising. However, no satisfactory treatment is currently available for this disease. Dementia is difficult to treat owing to its progressive and irreversible pathology in the brain, accompanied with mild memory loss in the early stages^1,2^. Therefore, intervention before the onset of symptoms is desirable^2–4^. Some clinical trials have shown that preventive nutritional supplements and physical exercise lower the incidence of dementia^2–4^. Particularly, food-derived compounds that improve cognitive function may be useful to safely prevent dementia. For example, astaxanthin, epigallocatechin, and resveratrol have prevented cognitive impairment^5–7^; however, the detailed underlying mechanisms remain unclear. Notably, the administration of a naturally occurring flavone, 7,8-dihydroxyflavone, ameliorated cognitive decline in dementia model mice^8,9^ possibly by activating the tropomyosin receptor kinase B (TrkB), which is a neurotrophin receptor and involved in enhancement of neuroplasticity, an essential process for learning and memory^10–13^.

The food-derived amino acid ergothioneine (ERGO) is not synthesized in the body but is orally absorbed from the daily diet in mammals. ERGO has a plasma baseline concentration of 1–3 µM in humans^14,15^. ERGO levels in the systemic circulation of patients with dementia are significantly lower than those in age-matched healthy subjects^16,17^ and are negatively correlated with neurodegeneration, including hippocampal atrophy^18^. Therefore, low ERGO levels may be a risk factor for dementia. Repeated oral administration of ERGO enhances learning and memory in both healthy and dementia mice models^19,20^. Moreover, the daily intake of food-extract tablets containing ERGO enhances verbal memory in humans, as demonstrated by the Cognitrax test, which evaluates cognitive function^21^. These findings imply that ERGO is essential for sustaining memory function, and ERGO supplements may have a beneficial effect on cognitive impairment. However, the detailed mechanisms of action of ERGO and the effect of its deficiency on cognitive function remain unknown.

Despite its hydrophilicity and low membrane permeability, ERGO is efficiently distributed to the brain following oral administration, primarily via the carnitine/organic cation transporter (OCTN1/SLC22A4), which is ubiquitously expressed in organs^22–24^. In brain parenchymal cells, OCTN1 is localized in the neurons, neural stem cells (NSCs), and microglia^24–26^ but not in astrocytes^27^. Oral administration of ERGO increased the number of mature spines in the hippocampus, and exposure to ERGO promoted synapse formation in cultured neurons of mice^19^. ERGO also promoted the neuronal differentiation of NSCs in primary cultures^28^, implying that OCTN1-mediated ERGO uptake may contribute to neural cell homeostasis. Notably, activation of neurotrophin/TrkB signaling has been proposed to be involved in these events^19,28^. Genetic polymorphisms in *TrkB* are associated with the onset of dementia^29,30^, and TrkB activation is considered a preventive strategy^9^. Thus, the enhancement of neurotrophin signaling by ERGO might promote neurogenesis and/or neuronal maturation, thereby affecting cognitive function. However, the involvement of neurotrophin signaling in promoting the beneficial effect of ERGO on cognitive impairment has not been investigated.

The purpose of the present study was to determine the fundamental role of ERGO in learning and memory by analyzing the effects of ERGO deficiency and supplementation on cognitive function in mice. Furthermore, we verified whether TrkB activation and hippocampal neurogenesis are the mechanisms underlying ERGO-induced cognitive function. It is also necessary to develop a diagnostic marker for the beneficial effect of ERGO to understand the cognitive enhancement and its mechanism in humans. As serum extracellular vesicles (EVs) contain brain-derived EVs, we utilized these to investigate the potential effect of ERGO on TrkB signaling and memory function in humans^22^. Phosphorylated TrkB (p-TrkB), the activated form of TrkB, was monitored in serum EVs derived from a previous clinical study in which the subjects were orally administered either ERGO-containing or placebo tablets^21^. We compared the p-TrkB levels in serum EVs with both exposure to ERGO in systemic circulation and ERGO-induced cognitive enhancement to reveal the possible involvement of TrkB in the enhancement of cognitive function in humans. In the present study, the ratio of p-TrkB to TrkB in serum EVs was proposed as a quantitative diagnostic marker of long-term ERGO-induced cognitive improvement.

## Results

### ERGO deficiency suppressed object recognition and location memory

To evaluate the effect of ERGO deficiency on learning and memory, ERGO-deficient mice were prepared by feeding an ERGO-free diet from 3 to 8 weeks of age. ERGO concentration in the plasma of the ERGO-deficient mice was below the quantification limit (<0.03 µM), whereas that in control mice fed with a normal diet was 1.24 ± 0.42 µM (Supplementary Fig. 1a). The hippocampal ERGO level in ERGO-deficient mice was 0.23 ± 0.03 nmol/g tissue, which was approximately nine times lower than that in control mice (2.00 ± 0.11 nmol/g tissue; Supplementary Fig. 1b). Learning and memory abilities were then assessed using the novel object recognition test (NORT) and spatial recognition test (SRT) at 8 weeks of age. In NORT, the exploration time for a novel object was significantly higher than that for a familiar object in control mice, whereas no significant difference in exploration time was observed between the novel and familiar objects in ERGO-deficient mice (Supplementary Fig. 1c). The discrimination index (DI), an indicator of learning and memory ability, was significantly lower in ERGO-deficient mice than in control mice (Supplementary Fig. 1d). In the SRT, the exploration time for a moved object was significantly higher than that for an unmoved object in control mice, whereas no significant difference in the exploration time was observed between the novel and familiar objects (Supplementary Fig. 1e). The DI in ERGO-deficient mice tended to be lower than that in control mice (Supplementary Fig. 1f).

**Fig. 1:**
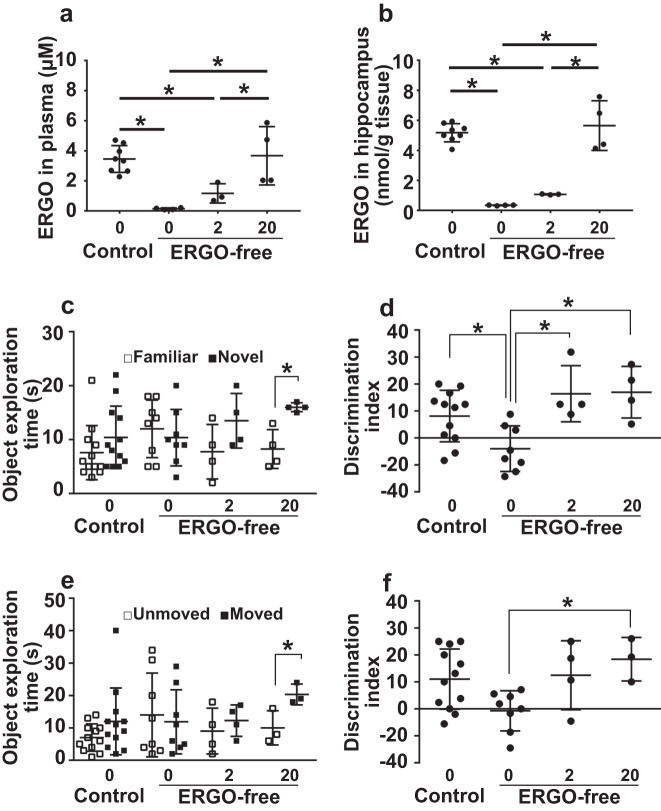
Recovery of learning and memory by oral ERGO administration in ERGO-deficient mice. Mice fed an ERGO-free diet were orally administered ERGO (0, 2, and 20 mg/kg) for two weeks, and ERGO concentration in plasma (**a**) and hippocampus (**b**) was measured. Mice fed the control diet were administered solvent alone. Each value represents the mean ± SD (*n* = 4–8). The statistical significance of the difference was determined by one-way ANOVA, followed by Tukey’s post hoc test (*P < 0.05). **c**–**f** Learning and memory ability was assessed via NORT (**c** and **d**) and SRT (**e** and **f**) after the ERGO treatment for two weeks. **c** Open and closed symbols represent exploration time for familiar and novel objects, respectively. **e** Open and closed symbols represent exploration time for unmoved and moved objects, respectively. **d**, **f** An indicator of learning and memory ability (DI) was calculated from the exploration time. Each value represents the mean ± SD (*n* = 3-12). *P < 0.05; one-way (**d** and **f**) or two-way (**c** and **e**) ANOVA followed by Tukey’s post hoc test.

To further examine the effects of ERGO on memory function, ERGO or vehicle alone was orally administered (three times per week for two weeks) to ERGO-deficient and control mice from nine weeks old to determine whether supplementation with ERGO recovered the decline in systemic ERGO concentration and memory function. ERGO-deficient mice were orally administered ERGO at 0, 2, and 20 mg/kg for two weeks and subjected to NORT and SRT, followed by the measurement of ERGO concentrations in the plasma and hippocampus. Oral administration of ERGO increased ERGO concentration in the plasma and hippocampus in a dose-dependent manner (Fig. 1a, b). We found that supplementation with 20 mg/kg of ERGO was sufficient to recover the hippocampal ERGO levels in ERGO-deficient mice to concentrations similar to that of control mice fed a normal diet (Fig. 1a, b). In the NORT, the exploration time for a novel object was significantly higher than that for a familiar object in ERGO-deficient mice administered 20 mg/kg ERGO (Fig. 1c). Administration of 2 and 20 mg/kg ERGO significantly increased the DI in ERGO-deficient mice (Fig. 1d). In the SRT, the exploration time for a moved object was significantly higher than that for an unmoved object in ERGO-deficient mice administered 20 mg/kg ERGO (Fig. 1e). Administration of 20 mg/kg ERGO significantly increased the DI in ERGO-deficient mice (Fig. 1f). Thus, oral supplementation with ERGO alone reversed the decline in object recognition and spatial memory in ERGO-deficient mice, suggesting that ERGO plays an essential role in these memory functions.

### Effect of ERGO deficiency and supplementation on neurogenesis and TrkB activation in the hippocampus

**Fig. 2:**
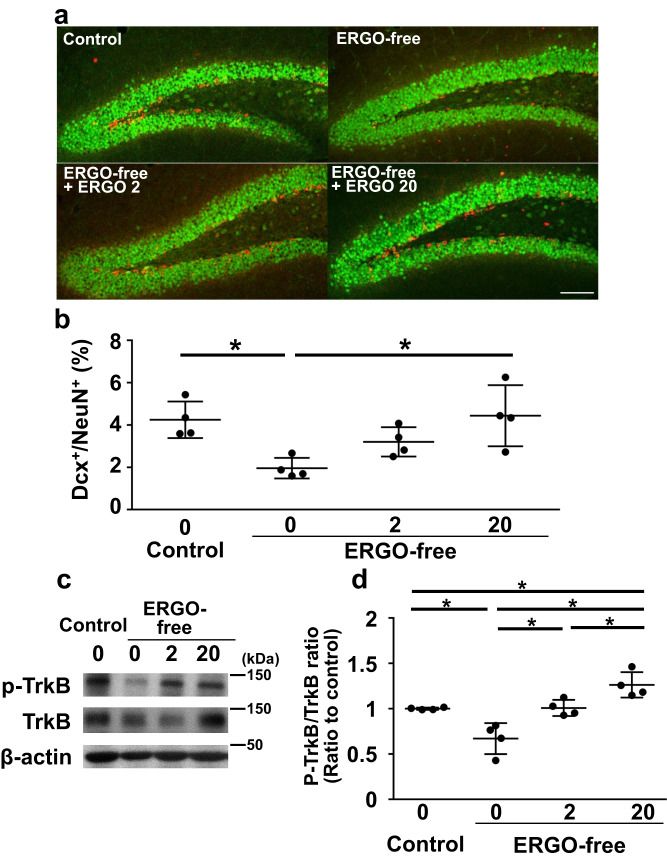
Recovery of hippocampal neurogenesis and TrkB phosphorylation by oral ERGO administration in ERGO-deficient mice. Mice fed an ERGO-free diet were orally administered ERGO (0, 2, and 20 mg/kg) for two weeks, and immunostaining and western blotting were performed for the hippocampus. Mice fed the control diet were administered solvent alone. **a** Images represent the expression of DCX (red) and NeuN (green) in the hippocampal dentate gyrus. Scale bar is 100 μm. **b** Percentage of the area occupied by Dcx^+^ cells from the total area occupied by NeuN^+^ cells was quantified using ImageJ. Each value represents the mean ± SD (*n* = 6-7). *P < 0.05; one way ANOVA followed by Tukey’s post hoc test. **c** p-TrkB, TrkB, and β-actin were detected in the hippocampal dentate gyrus via western blotting. Typical samples were shown. **d** The protein levels were measured using ImageJ software. Intensity of each band of p-TrkB was normalized by that of TrkB. Each value represents the mean ± SD (*n* = 4). *P < 0.05; one way ANOVA followed by Tukey’s post hoc test.

ERGO promotes synapse formation in neurons^19^ and neuronal differentiation of NSCs in primary culture*s*^28^; however, no evidence is available on its stimulatory effect on neurogenesis in vivo. Therefore, immunohistochemical analysis was performed in the hippocampal dentate gyrus following the final administration of ERGO to evaluate its effect on neurogenesis (Fig. 2a, b). The number of the newborn neuron marker Dcx-positive (Dcx^+^) cells in ERGO-deficient mice administered with vehicle alone appeared to be lower than that in control mice administered with vehicle alone, and administration of 2 and 20 mg/kg of ERGO increased the number in ERGO-deficient mice (Fig. 2a). Quantitative analysis revealed that the percentage of the area of Dcx^+^ cells in the neuronal nuclei marker NeuN-positive (NeuN^+^) cells in ERGO-deficient mice was significantly lower than that in control mice, and 20 mg/kg ERGO administration significantly increased the percentage, while 2 mg/kg of ERGO tended to increase it (Fig. 2b). Regarding hippocampal neurogenesis, the expression of phosphorylated TrkB (p-TrkB), an activated form of TrkB, was significantly lower in the dentate gyrus of ERGO-deficient mice administered with vehicle alone than in the dentate gyrus of control mice administered with vehicle alone, and ERGO administration significantly increased p-TrkB expression in a dose-dependent manner in ERGO-deficient mice (Fig. 2c, d). No difference was observed in the expression of total TrkB among all groups (Fig. 2c).

### Concomitant increase in ERGO-induced hippocampal neurogenesis and memory functions

**Fig. 3:**
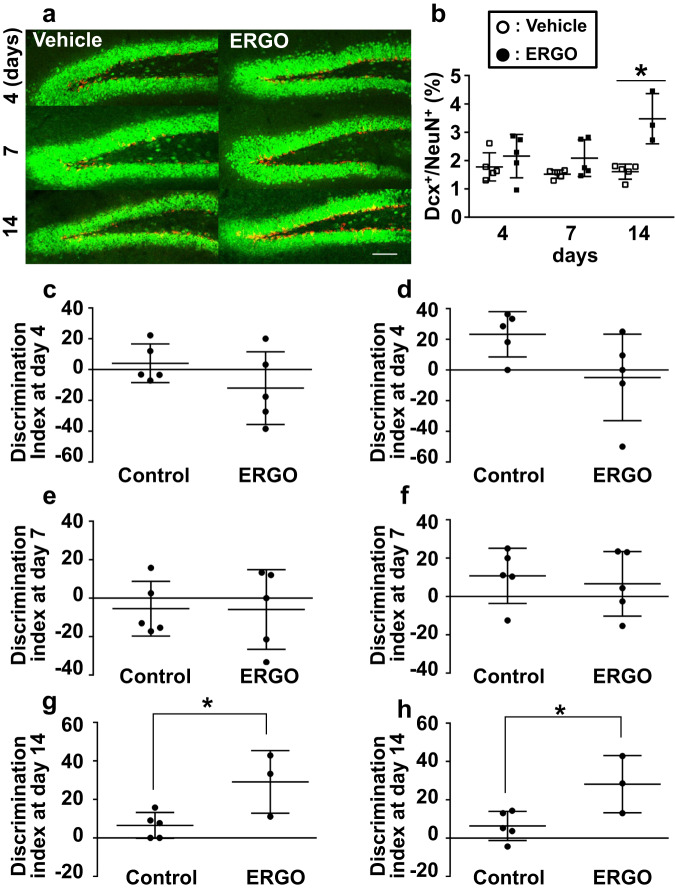
Time-dependent hippocampal neurogenesis and increase in cognitive function during oral administration of ERGO. Mice fed an ERGO-free diet were orally administered ERGO (50 mg/kg) or vehicle alone for 4, 7, or 14 days, followed by immunohistochemical analysis and behavior tests. **a** Images represent the expression of DCX (red) and NeuN (green) in the hippocampal dentate gyrus. Scale bar is 100 μm. **b** Percentage of the area occupied by Dcx^+^ cells from the total area occupied by NeuN^+^ was quantified using ImageJ. Each value represents the mean ± SD (*n* = 3–5). *P < 0.05; two-way repeated measures ANOVA followed by Tukey’s post hoc test. Learning and memory ability were evaluated via NORT (**c**, **e**, and **g**) and SRT (**d**, **f**, and **h**) at day 4 (**c** and **d**), 7 (**e** and **f**), and 14 (**g** and **h**). An indicator of learning and memory ability (DI) was calculated from the exploration time shown in Supplementary Fig. 2. Each value represents the mean ± SD (*n* = 3–5). *P < 0.05; Student’s *t* test.

Neurogenesis generally involves the proliferation of NSCs and their differentiation into neurons, which is a slow process. Therefore, to understand the possible relevance of ERGO-induced neurogenesis and memory function, we examined the increase after the start of ERGO administration at different time points. The percentage of area occupied by Dcx^+^ cells from the total area occupied by NeuN^+^ increased only two weeks after the start of ERGO administration, whereas no increase was observed at 4 or 7 days (Fig. 3a, b). In NORT, ERGO administration significantly increased DI only two weeks after the start of ERGO administration, whereas DI did not change at 4 or 7 days (Fig. 3c, e, g; Supplementary Fig. 2a, c, e). Similarly, the neurogenic effect of ERGO on DI in SRT was not observed at 4 or 7 days but was observed two weeks after the start of ERGO administration (Fig. 3d, f, h; Supplementary Fig. 2b, d, f). In addition, ERGO significantly increased the expression of p-TrkB normalized by total TrkB in hippocampal dentate gyrus only 14 days after the start of administration (Supplementary Fig. 3). Thus, two weeks of treatment with ERGO seemed to be necessary to induce enhancement of phosphorylation of TrkB, hippocampal neurogenesis, and cognitive function.

### Administration of TrkB inhibitor suppressed ERGO-induced enhancement of neurogenesis and memory function

**Fig. 4:**
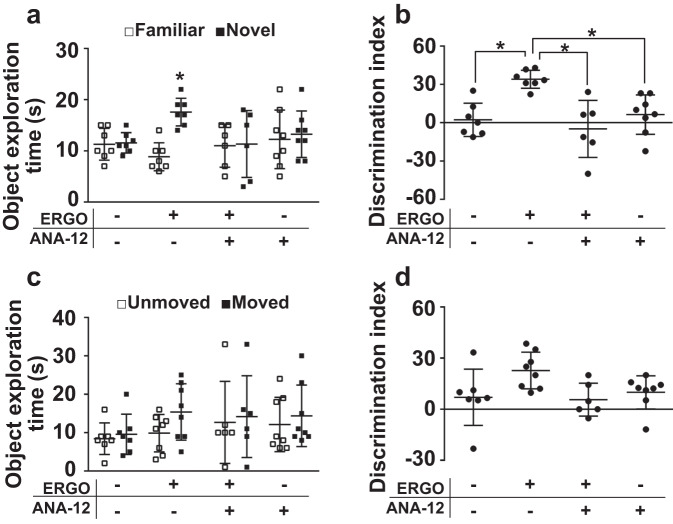
TrkB inhibitor suppressed ERGO-induced enhancement of memory function. **a**, **d** Mice fed an ERGO-free diet were orally administered ERGO (50 mg/kg) or vehicle alone with or without a TrkB inhibitor ANA-12. Learning and memory ability were assessed via NORT (**a** and **b**) and SRT (**c** and **d**) after the ERGO treatment for two weeks. **a** Open and closed symbols represent exploration time for familiar and novel objects, respectively. **c** Open and closed symbols represent exploration time for unmoved and moved objects, respectively. **b**, **d** An indicator of learning and memory ability (DI) was calculated from the exploration time. Each value represents the mean ± SD (*n* = 6–8). *P < 0.05; one-way (**b** and **d**) or two-way (**a** and **c**) ANOVA followed by Tukey’s post hoc test.

**Fig. 5:**
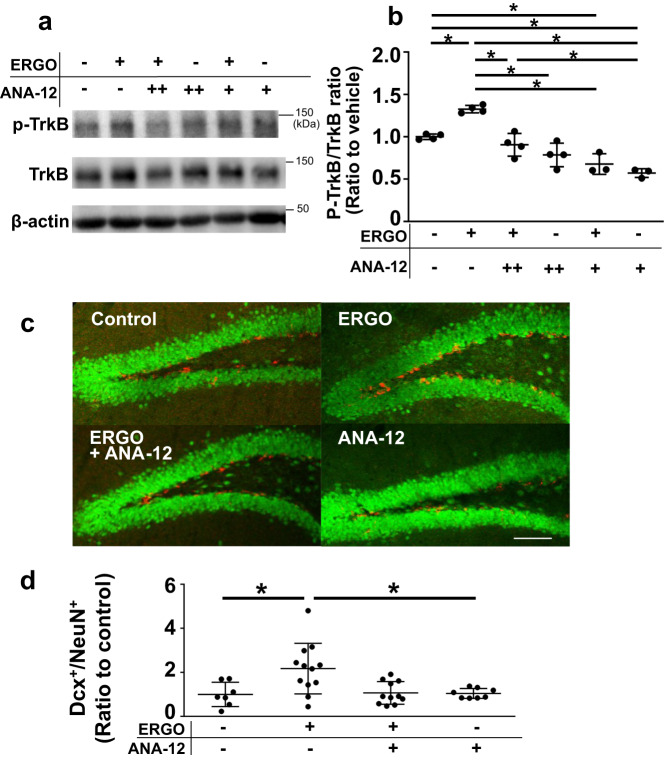
TrkB inhibitor suppressed ERGO-induced neurogenesis. **a**, **b** Western blot analysis was performed for the lysates of the hippocampal dentate gyrus in mice fed an ERGO-free diet, which were orally administered ERGO (50 mg/kg) or vehicle alone with or without a TrkB inhibitor ANA-12. **a** p-TrkB, TrkB, and β-actin in the typical samples are shown. **b** Those protein levels were measured using ImageJ software. Intensity of each band of p-TrkB was normalized by that of TrkB. Each value represents the mean ± SD (*n* = 3-4). *P < 0.05; one way ANOVA followed by Tukey’s post hoc test. **c**, **d** Immunohistochemical analysis was performed for the hippocampus of mice fed ERGO-free diet, which were orally administered ERGO or vehicle alone with or without ANA-12. **c** Images represent expression of DCX (red) and NeuN (green) in the hippocampal dentate gyrus. Scale bar is 100 μm. **d** Percentage of area occupied by Dcx^+^ cells from the total area occupied by NeuN^+^ was quantified using ImageJ. Each value represents the mean ± SD (*n* = 7–12). *P < 0.05; one way ANOVA followed by Tukey’s post hoc test. −, no treatment; +, 6 times per two weeks on days 0, 2, 4, 7, 9, and 11; ++, daily treatments.

To examine the involvement of TrkB in ERGO-induced enhancement of neurogenesis and memory function, ERGO-deficient mice were orally administered ERGO with or without a TrkB inhibitor, ANA-12, for 2 weeks. NORT showed that the exploration time for the novel object was significantly longer than that for the familiar object in the ERGO-treated group, while no difference between the novel and familiar objects was observed between the ERGO- and ANA-12 treated groups (Fig. 4a). ERGO also increased the DI of NORT, and ANA-12 suppressed this increase in DI induced by ERGO treatment (Fig. 4b). SRT showed that the exploration time for the moved object increased in the ERGO-treated group (Fig. 4c). ERGO also increased the DI of SRT; however, this increase was not observed when ANA-12 was co-administered (Fig. 4d). ERGO increased the ratio of p-TrkB to TrkB (p-TrkB/TrkB) in the dentate gyrus, while ANA-12 administration suppressed this increase (Fig. 5a, b). Thus, administration of ANA-12 was sufficient to inhibit ERGO-induced TrkB phosphorylation. The effect of TrkB inhibition on hippocampal neurogenesis was examined using immunohistochemical analysis. Administration of ERGO increased the percentage of area occupied by Dcx^+^ cells from the total area occupied by NeuN^+^, whereas the effect of ERGO on neurogenesis was not observed when ANA-12 was co-administered (Fig. 5c, d).

### Oral administration of ERGO-containing tablets increased serum concentrations of ERGO and the ratio of p-TrkB to TrkB in serum EVs in humans

In our previous clinical study, 5 mg ERGO-containing tablets prepared from food-extract or placebo tablets were orally administered healthy volunteers and subjects with mild cognitive impairment (MCI) once per day for 12 weeks, and ERGO concentrations in blood and Cognitrax memory functions were examined at weeks 0, 4, 8, and 12^21^. In the present study, serum concentrations of ERGO and its putative metabolites, S-methyl ERGO and hercynine, were measured in the same samples. ERGO and S-methyl ERGO concentrations at weeks 4, 8, and 12 were significantly higher than those at week 0 in the ERGO-treated group, whereas serum concentrations of S-methyl ERGO and hercynine were much lower than those of ERGO in both the ERGO-treated and placebo groups (Supplementary Fig. 4a–c). Blood ERGO concentration at week 12 in the ERGO-treated group (114 ± 54 µg/mL) was significantly higher than that in the placebo group (72.1 ± 42.1 µg/mL). These blood ERGO concentrations were ~100 times higher than the serum concentration (Supplementary Fig. 4).

**Fig. 6:**
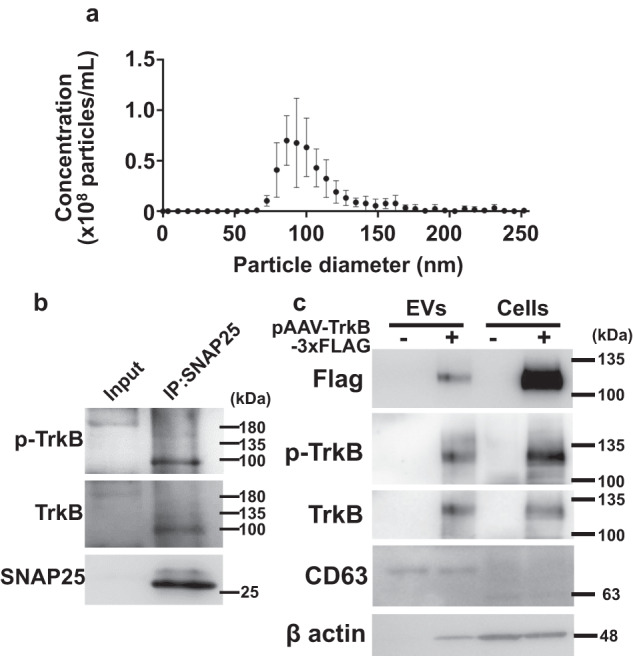
Characterization of EVs isolated from human serum. **a** The particle size distribution of serum EVs was analyzed using qNano. Each value represents the mean ± SD (*n* = 4). **b** Serum EVs were immunoprecipitated using anti-SNAP25 antibody, followed by western blotting with antibodies for p-TrkB, TrkB, and SNAP25. Serum EVs without immunoprecipitation was also subjected to western blotting and shown as input. **c** Neuro2A cells were transfected with pAAV-TrkB-3xFlag (+) or pAAV-tdTomato (−), and both EVs isolated from the culture medium and cell lysates were subjected to western blotting with antibodies for flag, p-TrkB, TrkB, CD63, and β-actin.

**Fig. 7:**
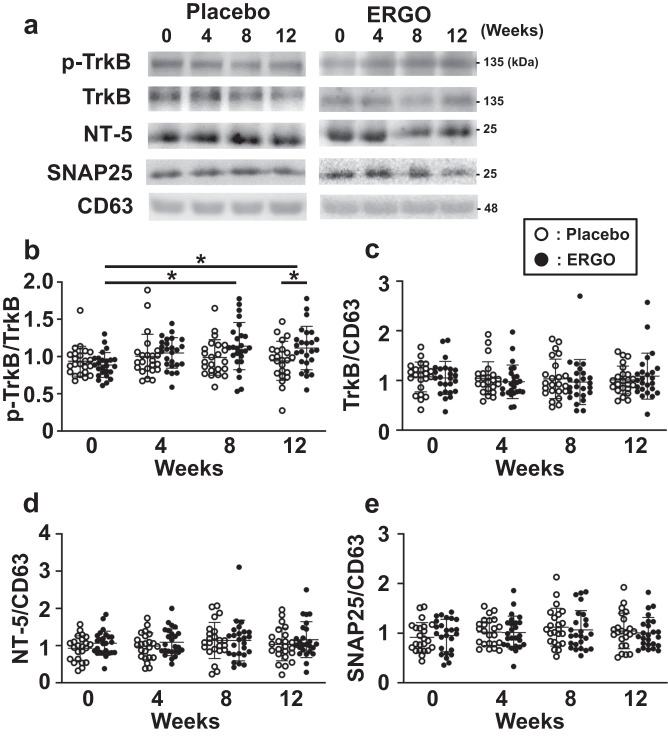
TrkB phosphorylation in serum EVs of humans after oral administration of ERGO-containing tablets was higher than that in the placebo group. **a** P-TrkB, TrkB, CD63, NT-5, and SNAP25 in the serum EVs of all participants were detected using western blotting. Typical samples are shown. **b**–**e** Protein levels were measured using ImageJ software. The intensity of each band of p-TrkB was normalized by that of TrkB (**b**), and the intensity of each band of TrkB (**c**), NT-5 (**d**), and SNAP25 (**e**) was normalized by that of CD63, followed by a comparison between the placebo (open symbol, *n* = 23) and ERGO-treated (closed symbol, *n* = 25) groups. Each value represents the mean ± SD. *P < 0.05; two-way repeated measures ANOVA followed by Tukey’s post hoc test.

To determine whether ERGO stimulates TrkB phosphorylation in humans, EVs were isolated from human serum samples, as they contain brain-derived EVs^31^. The average diameter of the EVs isolated from the control serum was 104 nm (Fig. 6a). The EVs obtained were then immunoprecipitated with antibodies against SNAP25, a marker for neuron-derived EVs, and p-TrkB and TrkB were detected in the immunoprecipitated EVs (Fig. 6b). We also confirmed that TrkB-expressing EVs were secreted from neuronal cells. EVs were isolated from the medium of Neuro2a cells transfected with TrkB-3xFlag, and both CD63, a marker for EVs, and TrkB-3xFlag were detected in the EVs of culture medium in TrkB-3xFlag transfected cells but not in cells transfected with tdTomato (Fig. 6c). EVs were isolated from all serum samples obtained from previous clinical studies^21^, and western blot analysis detected CD63 and SNAP25 (Fig. 7a), confirming that brain-derived EVs were included in the isolated EVs. The p-TrkB/TrkB ratio in the ERGO-treated group was significantly higher than that in the placebo group at week 12 (Fig. 7b). Furthermore, in the ERGO-treated group, p-TrkB/TrkB at weeks 8 and 12 was significantly higher than that at week 0 (Fig. 7b). In contrast, the administration of ERGO-containing tablets did not affect the expression of TrkB, NT-5 (a TrkB ligand), or SNAP25 normalized by the expression of CD63 (TrkB/CD63, NT-5/CD63, or SNAP25/CD63, respectively) (Fig. 7c–e).

### Correlation of p-TrkB/TrkB in serum EVs with systemic ERGO concentration and cognitive function in humans

**Fig. 8:**
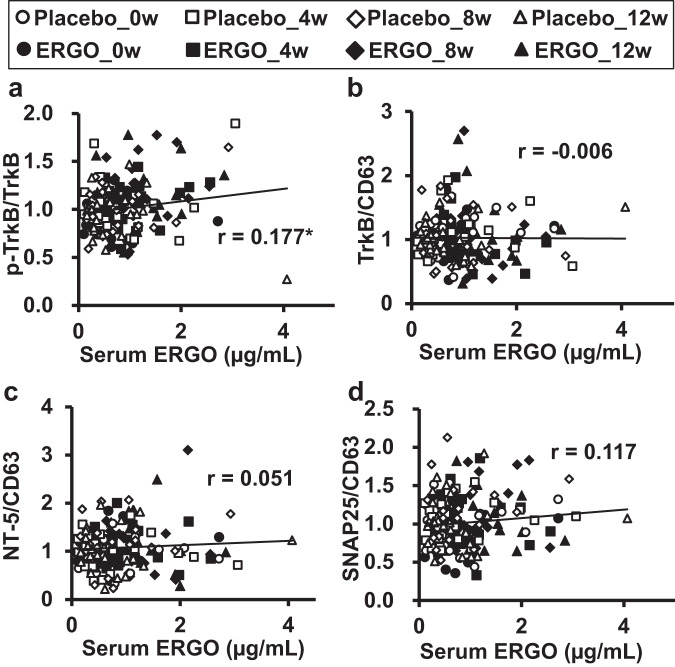
Scatter plots showing the relationship between serum ERGO concentration and ratio of each protein expression level in human serum EVs. **a** p-TrkB/TrkB, **b** TrkB/CD63, **c** NT-5/CD63, and **d** SNAP25/CD63. The plots included all data of human serum samples, serum EVs, and a cognitive domain score of Cognitrax obtained at weeks 0, 4, 8, and 12 after the start of oral administration of ERGO-containing and placebo tablets (placebo, *n* = 23; ERGO, *n* = 25). *P < 0.05; correlations were assessed via Pearson’s correlation coefficient. *r*, correlation coefficient.

Correlations between protein expression in serum EVs (p-TrkB/TrkB, TrkB/CD63, NT-5/CD63, and SNAP25/CD63) and serum ERGO concentration were first assessed using Pearson’s correlation analysis (Fig. 8a–d). Serum ERGO concentrations were significantly correlated with the p-TrkB/TrkB ratio (Fig. 8a) but not with TrkB/CD63, NT-5/CD63, or SNAP25/CD63 (Fig. 8b–d) when all serum EVs samples from the ERGO-treated and placebo groups were included. A significant correlation was also observed between the blood ERGO concentration and p-TrkB/TrkB (Supplementary Fig. 5a) but not between TrkB/CD63, NT-5/CD63, or SNAP25/CD63 (Supplementary Fig. 5b-d). The ΔAUC of serum ERGO, which represents an increase in systemic exposure to ERGO by oral administration of ERGO-containing tablets, was also significantly correlated with composite memory, verbal memory, and processing speed in the ERGO-treated group (Table 1), implying that ERGO may be associated with the enhancement of these cognitive functions.

**Table 1:** Correlation between each cognitive domain score of Cognitrax and the ratio of each protein expression level in serum EVs or ERGO ΔAUC in the group treated with ERGO-containing tablets^a^.

	p-TrkB/TrkB		TrkB/CD63		NT-5/CD63		SNAP25/CD63		ΔAUC for Serum ERGO^b^	
	r^c^	*P*^d^	r^c^	*P*^d^	r^c^	*P*^d^	r^c^	*P*^d^	r^c^	*P*^d^
Composite memory^e^	0.286	<0.01	−0.054	0.591	0.055	0.590	−0.023	0.823	0.244	<0.05
Verbal memory^e^	0.215	<0.05	−0.032	0.752	0.133	0.186	−0.015	0.884	0.240	<0.05
Processing speed^e^	0.204	<0.05	0.003	0.979	0.023	0.823	−0.045	0.660	0.240	<0.05
Visual memory^e^	0.235	<0.05	−0.053	0.599	−0.040	0.695	−0.021	0.838	0.187	0.108
Psychomotor speed^e^	0.113	0.264	0.073	0.469	−0.067	0.509	−0.080	0.428	0.105	0.371
Reaction time^e^	−0.284	<0.01	−0.120	0.234	0.057	0.572	−0.112	0.266	0.156	0.183
Complex attention^e^	−0.265	<0.01	0.117	0.246	0.011	0.914	0.111	0.272	−0.042	0.721
Cognitive flexibility^e^	0.332	<0.01	−0.087	0.388	−0.003	0.980	−0.054	0.596	0.064	0.586
Executive function^e^	0.335	<0.01	−0.086	0.397	−0.003	0.975	−0.051	0.616	0.060	0.607
Working memory^e^	0.256	<0.05	−0.026	0.797	0.011	0.914	−0.013	0.897	0.167	0.152
Sustained attention^e^	0.176	0.080	−0.066	0.516	−0.118	0.243	−0.115	0.255	0.098	0.403
Simple attention^e^	0.118	0.243	−0.084	0.406	0.007	0.945	−0.095	0.349	−0.043	0.713
Motor speed^e^	−0.006	0.951	0.107	0.287	−0.099	0.330	−0.063	0.533	−0.046	0.694

^a^Correlations were assessed using Pearson’s correlation coefficient.

^b^The area under the serum concentration curve (AUC) for ERGO subtracted from that of the baseline obtained at week 0.

^c^Correlation coefficient.

^d^Statistics *p*-values.

^e^Cognitive domain scores of Cognitrax. The higher score of all Cognitrax domains, except reaction time and complex attention, indicated a higher score for each function.

Correlations between the protein expression in serum EVs and each domain score in Cognitrax were assessed (Table 1). Among the domains of Cognitrax, composite memory, verbal memory, processing speed, visual memory, cognitive flexibility, excessive function, and working memory were positively correlated with p-TrkB/TrkB, whereas reaction time and complex attention were negatively correlated with p-TrkB/TrkB in the ERGO-treated group (Table 1), suggesting an association between p-TrkB/TrkB and cognitive function. In contrast, NT-5/CD63 and SNAP25/CD63 were not correlated with any Cognitrax domains (Table 1).

The correlation with serum EV proteins was also examined using the Mini Mental State Examination (MMSE) score, a test to evaluate cognitive function, before ERGO intervention. p-TrkB/TrkB was not correlated with MMSE score and was almost comparable between healthy controls (HC) and MCI before ERGO intervention (Supplementary Fig. 6a), suggesting that p-TrkB/TrkB may not reflect cognitive function without intervention. In contrast, both NT-5/CD63 and SNAP25/CD63 were significantly correlated with the MMSE score and were significantly higher in the HC group than in the MCI group (Supplementary Fig. 6c, d). This was consistent with a previous report that the expression of SNAP25 in circulating EVs discriminates cognitive function among healthy, mild cognitive impairment (MCI), and Alzheimer’s disease (AD)^31^, whereas no report is available for NT-5/CD63.

## Discussion

The present study demonstrated that an ERGO-free diet causes impairments in both hippocampal neurogenesis and cognition, accompanied by decreases in plasma ERGO levels and phosphorylation of TrkB in the hippocampus (Supplementary Fig. 1 and Fig. 2). Moreover, oral supplementation of ERGO alone recovered these impairments, with the concomitant restoration of ERGO levels in the plasma and hippocampus in mice (Fig. 1). This suggests that ERGO levels in the body may play an essential role in sustaining cognitive function, and supplementation with ERGO would be beneficial as a preventive therapy for reduced cognitive function, at least in rodents. In humans, blood ERGO levels in subjects with dementia were reported to be lower than those in normal subjects of the same age^16,17^, whereas ERGO-containing tablets enhanced cognitive function in healthy participants^21^. Moreover, a positive correlation between plasma ERGO levels and hippocampal volume has been reported in patients^18^. ERGO is not synthesized in mammals but is ingested from the daily diet, orally absorbed, and taken up by neuronal cells via its specific transporter OCTN1/SLC22A4^22,23^, implying its importance in brain function.

The present study also detected p-TrkB in serum EVs of humans, revealing that the p-TrkB/TrkB ratio in serum EVs in the group administered ERGO-containing tablets was significantly higher than that in the placebo group (Fig. 7b), and that p-TrkB/TrkB ratio in the serum EVs was positively correlated with both the systemic levels of ERGO and cognitive enhancement after the administration ERGO-containing tablets in humans (Table 1). These findings indicate that TrkB phosphorylation is associated with ERGO-induced enhancement of cognitive function in humans. The present study further focused on TrkB phosphorylation as a possible mechanism underlying ERGO-induced enhancement of cognitive function; ERGO-free diet decreased both TrkB phosphorylation and neurogenesis in the hippocampus, whereas oral supplementation with ERGO reversed these effects (Fig. 2a–d). This is consistent with a previous report that ERGO promotes neuronal differentiation of NSCs in primary culture via S6K1/NT-5/TrkB signaling^28^. It took two weeks for ERGO-induced TrkB phosphorylation, hippocampal neurogenesis, and cognitive enhancement (Fig. 3 and Supplementary Fig. 3), which may be due to the time-consuming process of brain distribution of ERGO and/or neurogenesis. Furthermore, the effects of ERGO were blocked by administering a TrkB inhibitor (Figs. 4 and 5). In addition, it has been reported that antioxidant activity of ERGO may not be involved in neuronal differentiation of NSCs^25^. These findings may suggest a pivotal role of TrkB phosphorylation in ERGO-induced enhancement of cognitive function and hippocampal neurogenesis. Activation of TrkB is crucial for neuroplasticity, such as neurogenesis and neuronal maturation^11,12^, which are essential for cognitive function^12,13^. Single nucleotide polymorphisms of *TrkB* were reported to be significantly associated with the onset of cognitive impairment in AD^29,30^. Additionally, TrkB activation ameliorates impairments in memory performance^8,9,32,33^, neurogenesis, and neuronal maturation in animal models of dementia^34,35^. All these previous reports suggest the role of TrkB as a target molecule for cognitive function improvement by ERGO.

In humans, the p-TrkB/TrkB ratio in serum EVs was significantly correlated with serum and blood ERGO levels (Fig. 8a and Supplementary Fig. 5a), and both composite and verbal memory scores were assessed in Cognitrax (Table 1). These results suggested that the p-TrkB/TrkB ratio in serum EVs quantitatively represents the cognitive efficacy of ERGO. Although a large number of biomarker candidates reflecting the pathology of neurodegenerative disorders have been proposed, only a few quantitative biomarkers have been established to monitor the effect of therapeutic agents on cognitive function in humans^31,36,37^. For example, the expression of synaptic proteins, such as SNAP25, GAP43, and neurogranin, in the plasma-derived EVs of patients with preclinical AD is lower than that in healthy controls^31^, and the expression of SNAP25 in serum-derived EVs is positively correlated with age- and education-corrected MMSE scores^36^. These results suggest the positive correlation between SNAP25/CD63 in serum EVs and MMSE scores in the participants before the intervention and are consistent with the present study (Supplementary Fig. 6d). We also focused on the neurotrophic factor NT-5, which is a TrkB ligand activating TrkB, because exposure of cultured NSCs to ERGO is reported to increase expression of NT-5, followed by promotion of neuronal differentiation^28^. Importantly, the NT-5/CD63 ratio in the serum EVs of patients with mild cognitive impairment was significantly lower than that in healthy volunteers, and the ratio was positively correlated with the MMSE score (Supplementary Fig. 6c). Thus, NT-5/CD63 in serum EVs might be useful for the diagnosis of mild cognitive impairment, as in the case of SNAP25.

In the present study, human serum EVs were immunoprecipitated using an antibody against the synaptic protein SNAP25, and both p-TrkB and TrkB were detected in the precipitated EVs (Fig. 6b), suggesting that TrkB-expressing EVs are at least partially derived from the central nervous system. The expression of TrkB in the brain is much higher than that in the peripheral tissues of both rodents and humans^38,39^. Therefore, it can be speculated that TrkB detected in human serum EVs may reflect TrkB expressed in the central nervous system. The p-TrkB/TrkB in mouse serum EVs would support the speculation, but has not yet been detected in our preliminary studies probably because of too little volume of serum samples. On the other hand, the speculation was further supported by the detection of Flag-tagged TrkB, TrkB, and p-TrkB in the culture medium of neuronal cells Neuro2a exogenously transfected with TrkB-3xFlag (Fig. 6c). However, the physiological roles of TrkB-loaded EVs in the circulation are still unknown. EVs derived from brain parenchymal cells may be washed out, at least partially, by cerebrospinal fluid in the systemic circulation and thereby cleared by peripheral organs. Serum EVs have been proposed to play physiological roles in the transfer of proteins, nucleic acids, and nutrients^40–42^. TrkB in adipose tissue has a substantial influence on feeding behavior and obesity in female mice^43^, and the activation of endothelial TrkB induces the relaxation of resistance arteries^44^. Further studies are required to investigate the potential roles of TrkB-loaded EVs derived from the brain in peripheral organ functions.

Altogether, oral supplementation with ERGO recovered impairments in hippocampal neurogenesis and cognition in mice fed an ERGO-free diet, possibly via phosphorylation of TrkB. This suggests a fundamental role of ERGO in brain function. Additionally, TrkB phosphorylation was detected in human serum EVs and was considered to be associated with ERGO-induced cognitive enhancement in humans.

## Methods

### Animals

Three-week-old male ICR mice were purchased from Sankyo Labo Service Co. (Toyama, Japan). Mice were fed an ERGO-free diet (Basal Diet 5755, PMI Nutrition International) to prepare ERGO-deficient mice, following a previously reported protocol^19^, whereas mice fed a normal diet (PicoLab® Rodent Diet 5053, PMI Nutrition International) were used as controls. Mice were kept in a room under pathogen-free conditions at a controlled temperature (21–25 °C) with a 12 h dark/light cycle and fed food and water *ad libitum*. To evaluate the effect of ERGO on cognitive decline, ERGO-deficient and normal mice at the age of 9 weeks were orally administered ERGO dissolved in sterile distilled water at 0, 2, or 20 mg/kg on days 0, 2, 4, 7, 9, and 11, followed by behavioral testing. To examine the effect of ANA-12, an inhibitor of TrkB, ERGO-deficient mice at the age of 6 weeks were orally administered ERGO (50 mg/kg) on days 0, 2, 4, 7, 9, and 11, and the mice were administered intraperitoneally with ANA-12 (0.5 mg/kg) or vehicle (1% DMSO) daily or 30 min before each administration of ERGO.

### Behavioral tests

NORT and SRT were performed to assess learning and memory abilities according to the method described by Nakamichi et al.^19^, with minor modifications. Fourteen days after the first administration of ERGO, NORT was performed. Each mouse was placed in an acrylic chamber (45 cm ×45 cm ×45 cm) without any objects and allowed to explore for 10 min. The following day, the mouse was placed in the same chamber with two identical objects located on a diagonal line and allowed to explore the chamber for five minutes. The time spent exploring each object was then recorded. Eight hours later, one of the objects was replaced with a novel object of a different shape at the same location in the chamber. Each mouse was allowed to explore the chamber for five minutes. The exploration time for each object was recorded. The DI, an indicator of learning and memory ability, was calculated as: [(novel object exploration time/total exploration time) − (familiar object exploration time/total exploration time)] × 100.

The SRT was conducted on the day following NORT. Each mouse was placed in an acrylic chamber (45 cm ×45 cm ×45 cm) without any objects and allowed to explore for 10 min. The next day, the mouse was placed in the same chamber with two identical objects located on a diagonal line and was allowed to explore the chamber for nine minutes. The time spent exploring each object was then recorded. One hour later, one of the objects was moved, and each mouse was allowed to explore the chamber for five minutes. The exploration time for each object was recorded. The DI was calculated as: [(moved object exploration time/total exploration time) − (unmoved object exploration time/total exploration time)] × 100.

In the experiment evaluating the effect of ANA-12, 8 h of retention time in the NORT was increased to 24 h, and 9 min of exploration time in the SRT was reduced to 5 min, without any change in other experimental conditions.

### Immunohistochemistry

After the behavioral tests, each mouse was anesthetized with isoflurane and subjected to transcardial perfusion with 5 mL PBS and 20 mL 4% paraformaldehyde in phosphate buffer. Each brain sample was isolated and incubated with 4% paraformaldehyde in phosphate buffer at 4 °C for 24 h. After washing with PBS, each brain was sectioned with a Neo-LinearSlicer (DOSAKA EM, Kyoto, Japan) at a thickness of 100 µm. The sections were incubated for 30 min in 0.1% Tween 20 in PBS (PBST) containing 3% bovine serum albumin (BSA) at room temperature. They were incubated with PBST containing goat anti-doublecortin (Dcx) antibody (1:500 dilution; sc-8066, goat, Santa Cruz; Dallas, TX, USA) and mouse anti-NeuN antibody (1:500 dilution; MAB377, mouse, Merck Millipore; Burlington, MA) overnight at room temperature, followed by washing with PBST and probing with anti-goat Alexa555 (1:500 dilution; A21432, donkey, Sigma-Aldrich; St. Louis, MO) and anti-mouse Alexa488 (1:500 dilution; A21202, donkey, Thermo Fisher Scientific; Waltham, MA) overnight at room temperature. The sections were rinsed again with PBS, treated with a mounting medium containing 2-(4-aminophenyl)-1H-indole-6-carboxamidine, and observed under an LSM710 confocal laser-scanning microscope (Carl Zeiss, Jena, Germany). The newborn neuron marker Dcx and the neuronal nuclei marker NeuN were colored red and green, respectively. Quantification was performed by measuring the immunoreactive area of Dcx or NeuN in double immunocytochemical analysis.

### Clinical study

A randomized, placebo-controlled, double-blind, parallel-group clinical study (UMIN000034386; placebo group, *n* = 23; group treated with ERGO-containing tablets, *n* = 25), including healthy volunteers and subjects with MCI, was conducted for 12 weeks, as previously described (Supplementary Fig. 7)^21^. Subjects were divided into two groups based on their age and MMSE scores. Participants in the ERGO-treated group were orally administered four food extract tablets containing 5 mg ERGO daily with water, whereas those in the placebo group were administered ERGO-free tablets in the same manner as the ERGO-treated group. Neither tablet could be distinguished by its appearance or smell. All participants were instructed to refrain from eating foods containing ERGO, such as mushrooms, during the study period. Serum collection and cognitive function tests (Cognitrax) were performed at weeks 0, 4, 8, and 12.

### Measurement of ERGO and its metabolites

Hippocampal samples were weighed and homogenized using a Precellys homogenizer (Bertin Technologies, Montigny-le-Bretonneux, France) in 12 volumes of methanol containing L-(+)-ergothioneine-d9 (ERGO-d9; Toronto Research Chemicals Inc., North York, ON) as an internal standard, whereas plasma and serum samples were mixed with nine volumes of methanol containing ERGO-d9. The mixtures were centrifuged (21,500 × *g*, 10 min, 4 °C) twice and subjected to liquid chromatography-mass spectrometry/mass spectrometry (LCMS-8040; Shimadzu, Kyoto, Japan) equipped with a Luna 3.0 µm HILIC column (200 Å, 150 × 2.0 mm; Phenomenex, Torrance, CA) to quantify ERGO, S-methyl-ERGO, and hercynine. Chromatography was performed using step-gradient elution (flow rate, 0.4 mL/min) as follows: The mobile phases were (A) 10 mM ammonium acetate/0.1% formic acid/80% water/20% acetonitrile and (B) 10 mM ammonium acetate/0.1% formic acid/5% water/95% acetonitrile. 0–0.5 min: 1% A/ 99% B; 0.5–1.9 min: 1% A/ 99% B to 20% A/ 80% B; 1.9–4.5 min: 20% A/ 80% B; 4.5–5.5 min: 20% A/ 80% B to 60% A/ 40% B; 5.5–6.5 min: 60% A/ 40% B; 6.5–6.7 min: 60% A/ 40% B to 1% A/ 99% B; 6.7–7.2 min: 1% A/ 99% B. Each compound was measured in the ESI positive mode (ERGO: 230.3 > 127.0, S-methyl-ERGO: 244.1 > 140.95, hercynine: 198.0 > 95.05, ERGO-d9: 239.2 > 127.0). The area under the serum concentration curve (AUC) of ERGO subtracted from the baseline level of ERGO at week 0 (ΔAUC) was calculated using the following Eq. (1):

1 $$\Delta {\rm{AUC}}=\mathop{\sum }\limits_{n=1}^{3}2\left({y}_{n-1}\,+\,{y}_{n}{-2y}_{0}\right)$$

where ${y}_{0}$, ${y}_{1}$, ${y}_{2}$, and ${y}_{3}$ represent the serum ERGO concentrations at weeks 0, 4, 8, and 12, respectively.

### Isolation of EVs from serum

One milliliter of serum was first centrifuged at 1200 × *g* for 20 min, and the supernatant was then centrifuged at 17,000 × *g* for 20 min. The collected supernatant was passed through a 0.22 µm filter, followed by ultracentrifugation at 100,000 × *g* for 90 min (TLS-55 Rotor, Optima MAX-TL, Beckman Coulter, Brea CA). The pellets were resuspended in 50 µL of phosphate-buffered saline (PBS) and used for each experiment.

### Nanoparticle analysis

The concentration and size distribution of EVs were measured with tunable resistive pulse sensing by qNano (Izon Science, UK) using an NP100 nanopore (Izon Science, UK), according to the method described by Lane et al.^45^ with minor modifications. All measurements were calibrated with 100 nm polystyrene beads (CPC100; Izon Science, UK). Data analysis was carried out using Izon Control Suite software v3.1 (Izon Science, UK).

### Isolation of brain-derived EVs via immunoprecipitation

Serum EVs were isolated via size exclusion chromatography according to the method described by Böing et al.^46^, with minor modifications. Briefly, the tip of a 10 mL plastic syringe (Terumo, Tokyo, Japan) was stuffed with nylon stocking (DAISO, Tokyo, Japan), and the syringe was stacked with 5 mL of washed Sepharose CL-2B (GE Healthcare, Uppsala, Sweden). After equilibration with PBS, 1 mL of serum was loaded onto the column and eluted with PBS. The eluate was collected into 25 sequential fractions (0.5 mL). The number and size of particles were determined using qNano for each fraction, and particles were detected in fractions 6–12. These fractions (3.5 mL) were combined and concentrated to approximately 200 µL using Amicon Ultra-15 (Merck Millipore, Burlington, MA, USA). To isolate brain-derived EVs, an antibody against the mature neuronal marker SNAP25 (1:25 dilution; sc-390644, mouse, Santa Cruz; Dallas, TX, USA) was first incubated with Protein G Sepharose 4 Fast Flow (Cytiva, Marlborough, MA, USA) in PBS containing 0.01% Triton-X 100 and phosphatase inhibitors (10 mM sodium fluoride, 10 mM β-glycerophosphate disodium salt hydrate, 10 mM sodium pyrophosphate decahydrate, and 1 mM sodium orthovanadate) at 4 °C for 2 h, followed by blocking with PBS containing 10% BSA and phosphatase inhibitors. The beads and EV samples were then mixed and incubated under rotation at 4 °C for 2 h. The samples were centrifuged at 15,000 × *g* for 2 min at 4 °C, and the supernatant was discarded. The pellets were washed twice with PBS containing 0.01% Triton-X 100 and phosphatase inhibitors, followed by western blotting as described below.

### Western blotting

Western blotting was performed according to the method described by Ishimoto et al.^28^, with minor modifications. Briefly, isolated hippocampal dentate gyrus and EVs were homogenized using a tip sonicator (TOMY SEIKO Co. Ltd., Tokyo, Japan) with 20 mM Tris–HCl buffer (pH 7.5) containing 1 mM EDTA, 1 mM EGTA, protease inhibitors (0.1 mM 4-(2-aminoethyl) benzenesulfonyl fluoride hydrochloride, 1 μg/mL leupeptin, 1 μg/mL antipain, and 0.5 mM benzamidine hydrochloride hydrate), and phosphatase inhibitors (10 mM sodium fluoride, 10 mM β-glycerophosphate disodium salt hydrate, 10 mM sodium pyrophosphate decahydrate, and 1 mM sodium orthovanadate), followed by protein concentration measurements with a Pierce^TM^ BCA Protein Assay Kit (ThermoFisher Scientific, Waltham, MA). The homogenates were added at a volume ratio of 1:4 to 10 mM Tris–HCl buffer (pH 6.8) containing 10% glycerol, 2% sodium dodecyl sulfate, 0.01% bromophenol blue, and 5% 2-mercaptoethanol, and the mixtures were incubated on a shaker at room temperature for 1 h, followed by incubation at 95 °C for 5 min. Each aliquot of 10 µg of protein from the hippocampal dentate gyrus was loaded onto a 10% polyacrylamide gel for electrophoresis at a constant current of 40 mA/plate for 90 min at room temperature using a PAGE system (Sima Biotech, Chiba, Japan). For EV samples, each aliquot of 20 µg of protein was loaded on a 12.5% polyacrylamide gel for electrophoresis at a constant current of 21 mA/plate for 30 min at room temperature using a compact-slab size PAGE system (ATTO, Tokyo, Japan), followed by blotting to a polyvinylidene fluoride membrane pretreated with 100% methanol. For blocking, BSA or skim milk dissolved in 20 mM Tris–HCl buffer (pH 7.5) containing 137 mM NaCl and 0.1% Tween 20 were used depending on the antibodies (2% BSA: p-TrkB, TrkB, NT-5, Flag, and β-actin; 1% skim milk: SNAP25; 3% skim milk: CD63). The membranes were probed with antibodies against TrkB (1:1,000 dilution; 4603 S, rabbit, Cell Signaling Technology; Danvers, MA), p-TrkB at Tyr816 (1:100 dilution; ABN1381, rabbit, Merck Millipore; Burlington, MA), NT-5 (1:250 dilution; AB1781SP, rabbit, Merck Millipore; Burlington, MA), SNAP25 (1:50 dilution; sc-390644, mouse, Santa Cruz; Dallas, TX), CD63 (1:100 dilution; sc-15363, rabbit, Santa Cruz; Dallas, TX), Flag (1:1,000 dilution; 14793S, rabbit, Cell Signaling Technology; Danvers, MA), and β-actin (1:10,000 dilution; A5441-.2 ML, mouse, Sigma-Aldrich; St. Louis, MO) diluted with Can Get Signal Solution 1 (TOYOBO, Osaka, Japan), followed by probing with a secondary anti-rabbit IgG antibody conjugated with peroxidase (1:2,000 dilution; 7074, goat, Cell Signaling Technology; Danvers, MA) or anti-mouse IgG antibody conjugated with peroxidase (1:10,000 dilution; A9917-1ML, goat, Sigma-Aldrich; St. Louis, MO) diluted with Can Get Signal Solution 2. Proteins that reacted with these antibodies were detected with ECL™ detection reagents using a lumino image analyzer (LAS-4000; FUJIFILM, Tokyo, Japan). Densitometric analysis of the western blots was performed using ImageJ software.

### Plasmid construction

pAAV-CMV-ZsGreen1 was purchased from Takara Bio. TdTomato was amplified from the pCSCMV:tdTomato plasmid (Addgene plasmid #30530; http://n2t.net/addgene:30530; RRID: Addgene_30530)^47^. Murine TrkB-3xFlag was amplified from the murine hippocampus using the DNA polymerase KOD FX NEO with the following sense and antisense primers: 5’-tgtaatcgatgtcatgatctttataatcaccgtcatggtctttgtagtccatCGCGCCTAGGATATCCAG-3’, 5’-tcacagggatgccacccgtggatccTCACTTGTCATCGTCATCCTTGTAATCGATGTCATGATCTTTATA-3’. The sequence encoding tdTomato in pAAV-CMV-tdTomato was replaced with TrkB-3xFlag using a NEBuilder HiFi DNA assembly kit (New England Biolabs).

### Cell culture

Neuro2a cells were cultured according to the method described by Nakamichi et al.^24^, with minor modifications. Briefly, Neuro2a cells were seeded at a density of 1.2 × 10^6^ cells/dish on φ10 cm dishes in DMEM supplemented with 100 units/mL penicillin, 100 µg/mL streptomycin, and 10% EV-depleted FBS, which was the supernatant after ultracentrifugation at 100,000 × g for 16 h at 4 °C. After 24 h, the cells were transiently transfected with pAAV-CMV-TrkB-3xFlag or pAAV-CMV-tdTomato using PEI MAX (Polysciences, Warrington, PA, USA), according to the manufacturer’s instructions, and the medium was changed to DMEM supplemented with 2% EV-depleted FBS and 20 µM retinoic acid for neuronal differentiation. At 48 h after seeding, the medium was changed to DMEM containing 100 nM of the TrkB agonist 7,8-dihydroxyflavone or vehicle. At 72 h after seeding, the cells and medium were collected. The cultures were maintained in a humidified atmosphere of 5% CO_2_/ 95% air at 37 °C.

### Statistics

Data were expressed as means ± standard deviation (SD). The statistical significance of the differences was determined using the Student’s *t* test or one-way or repeated measures ANOVA, followed by Tukey’s post hoc test. Correlations were determined using Pearson’s correlation coefficient after the Kolmogorov–Smirnov test. Statistical analyses were performed using Prism 7 (GraphPad Software, San Diego, CA, USA) and IBM-SPSS version 25.

### Study approval

Animal studies were approved by the Committee on the Ethics of Animal Experiments of the University of Kanazawa (permit number: AP-183968) to minimize animal suffering and loss of life. The clinical studies were conducted following the Declaration of Helsinki and approved by the Institutional Review Boards of Kanazawa University and the Japan Food Evidence Association. All participants provided written informed consent for the use of their samples and medical information for research purposes.

### Reporting summary

Further information on research design is available in the Nature Research Reporting Summary linked to this article.

### Supplementary information

Supplementary Material

Reporting summary

## Data Availability

The data supporting the findings reported herein are available on request from the corresponding author.
