# Supplementary material for: TrkB phosphorylation in serum extracellular vesicles correlates with cognitive function enhanced by ergothioneine in humans
Source: NPJ Sci Food. 2024 Feb 6;8:11. doi: 10.1038/s41538-024-00250-5 (PMC10847428; doi:10.1038/s41538-024-00250-5)
Supplement: Supplementary file 1 — Supplementary Material [file 41538_2024_250_MOESM1_ESM.pdf]

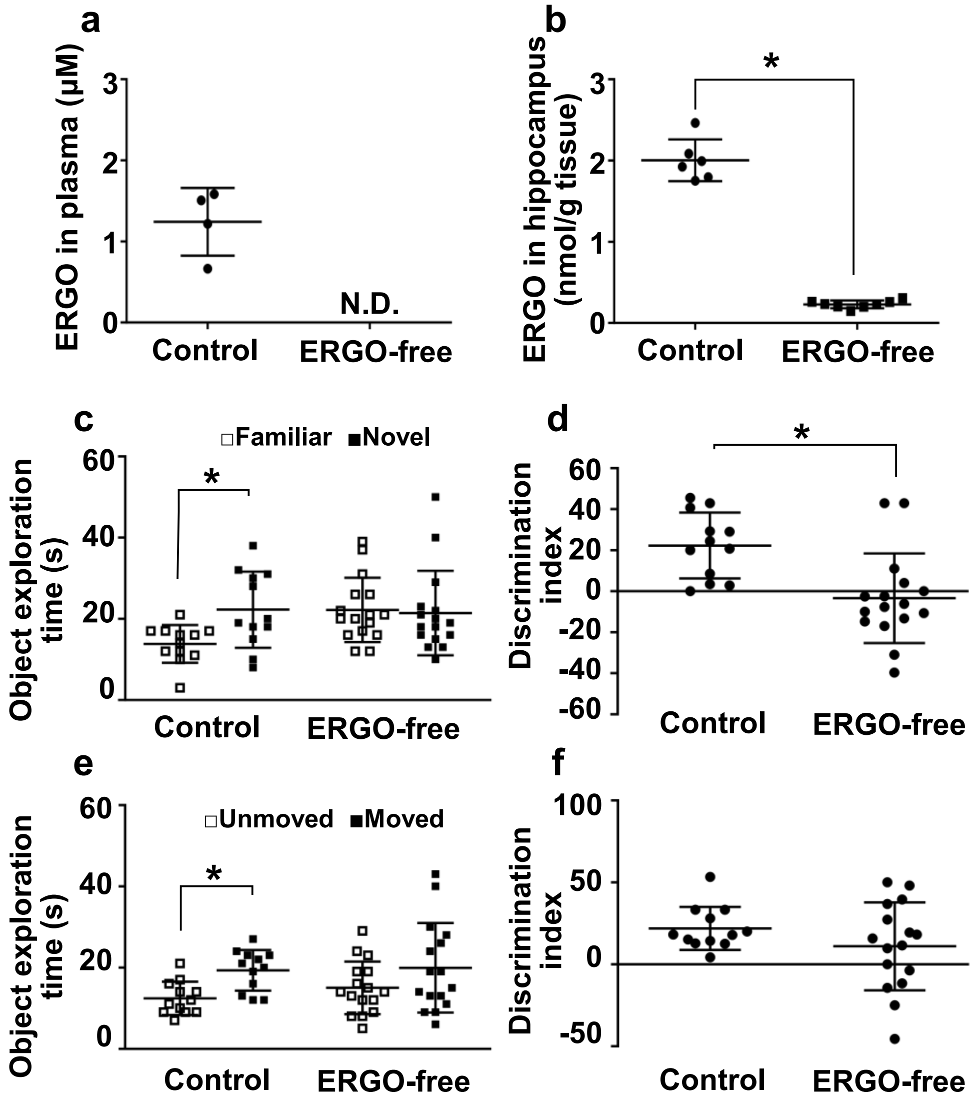

Supplemental Figure 1. Comparison of learning and memory in mice fed control and ERGO-free diet. ERGO concentration in the plasma (a) and hippocampus (b) of mice fed an ERGO-free diet and normal diet without ERGO administration (control) at 8 weeks of age. Each value represents the mean ± SD (n = 4-8). The statistical significance of differences was determined using Student's t-test. **P* < 0.05. N.D., not detectable. Learning and memory ability were assessed using a novel object recognition test (NORT, c, and d) and a spatial recognition

test (SRT, e, and f) at 8 weeks of age. In panel (c), open symbols are the exploration times for familiar objects, and closed symbols are those for novel objects. In panel (e), open symbols are the exploration times for the unmoved object, and closed symbols are those for the moved object. DI is an indicator of learning and memory ability calculated from these exploration times. Each value represents the mean ± SD (n = 12-16). *$P < 0.05$; two-way ANOVA followed by Tukey's post-hoc test. N.D., not detectable.

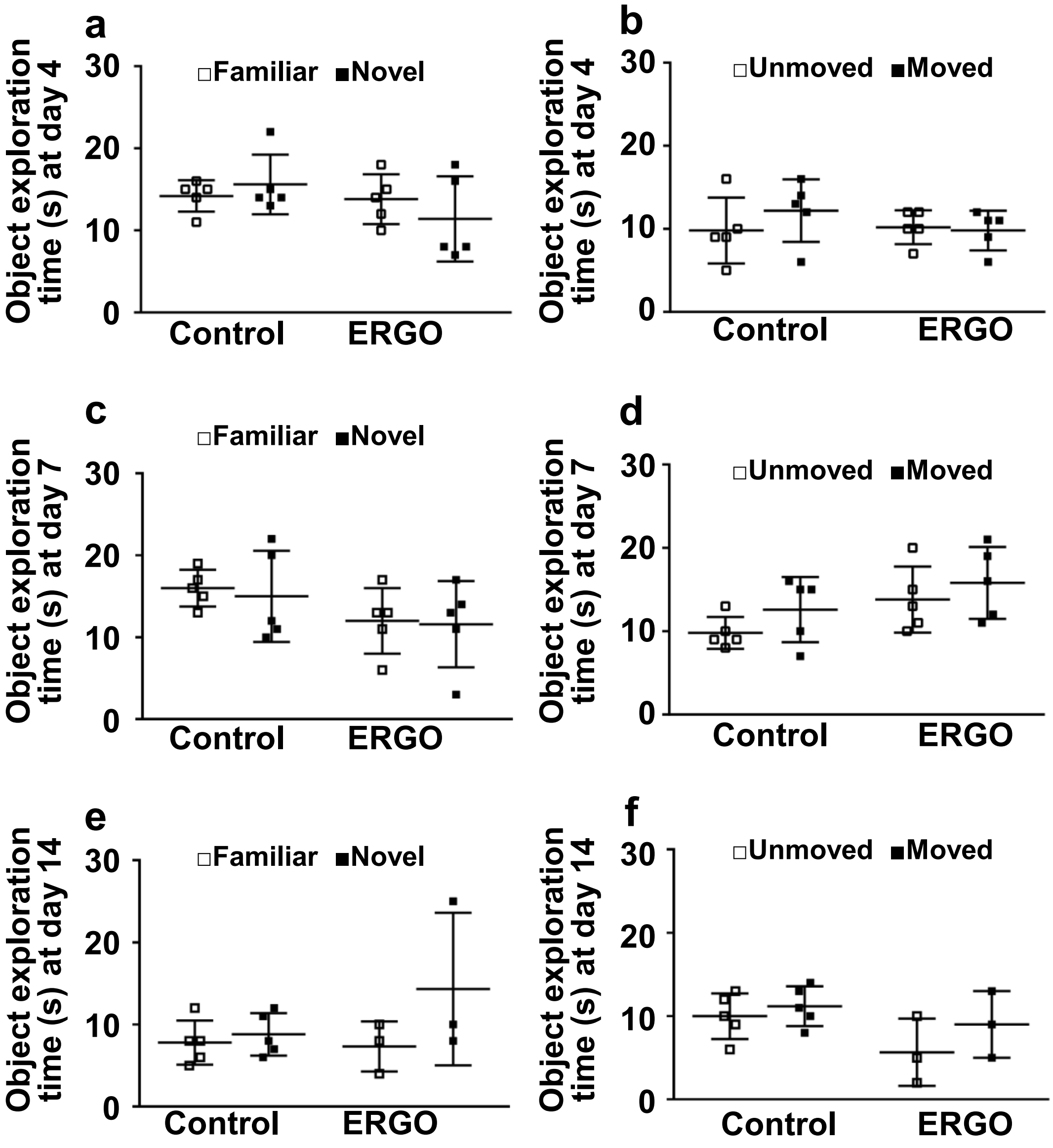

Supplementary Figure 2. Time-dependent increase in cognitive function after oral administration of ERGO. Mice fed ERGO-free diet were orally administered ERGO (50 mg/kg) or vehicle alone for 4 (a and b), 7 (c and d), and 14 days (e and f), followed by evaluation of learning and memory ability via NORT and SRT. Open and closed symbols represent the exploration time for familiar (or unmoved) and novel (or moved) objects, respectively. An indicator of learning and memory ability (DI) was calculated from these data

and shown in Figure 3. Each value represents the mean ± SD (n = 3-5). $^*P < 0.05$; two-way ANOVA followed by Tukey’s post-hoc test

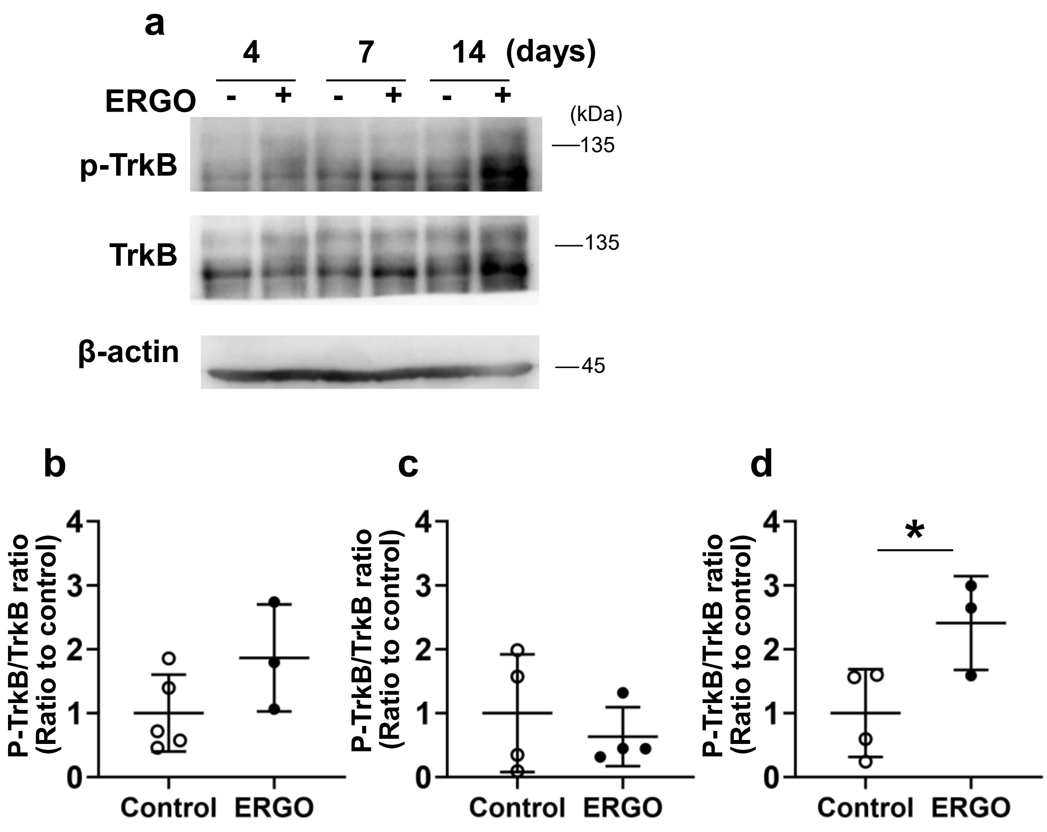

Supplemental Figure 3. Time-dependent increase in phosphorylation of TrkB in hippocampal dentate gyrus after oral administration of ERGO. Mice fed an ERGO-free diet were orally administered ERGO (50 mg/kg) or vehicle alone for 4, 7, or 14 days, followed by western blot analysis. (a) Typical blots of p-TrkB, TrkB, and β-actin are shown. Those protein levels at day 4 (b), 7 (c), and 14 (d) were measured using ImageJ software. Intensity of each band of p-TrkB was normalized by that of TrkB. Each value represents the mean ± SD (n = 3-5). *$P < 0.05$; Student's t-test.

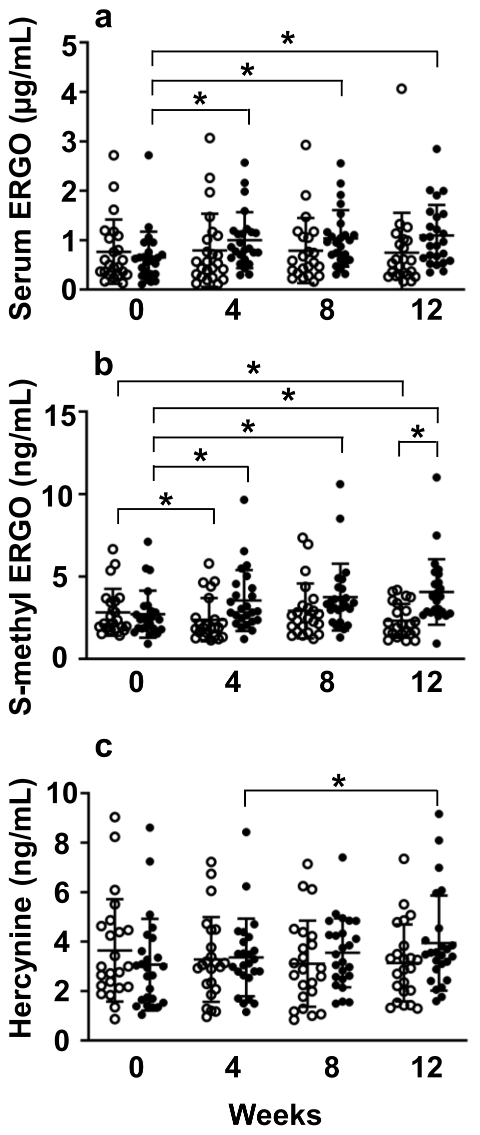

Supplemental Figure 4. Serum concentration of ERGO and its putative metabolites in healthy subjects orally administered ERGO-containing or placebo tablets. ERGO (a), s-methyl ERGO (b), and hercynine (c) in the serum were quantified using LC-MS/MS (open symbol: placebo, closed symbol: ERGO-containing tablets). Each value represents the mean ± SD (placebo, n = 23; ERGO, n = 25). *$P < 0.05$; two-way repeated measures ANOVA followed by Tukey's post hoc test.

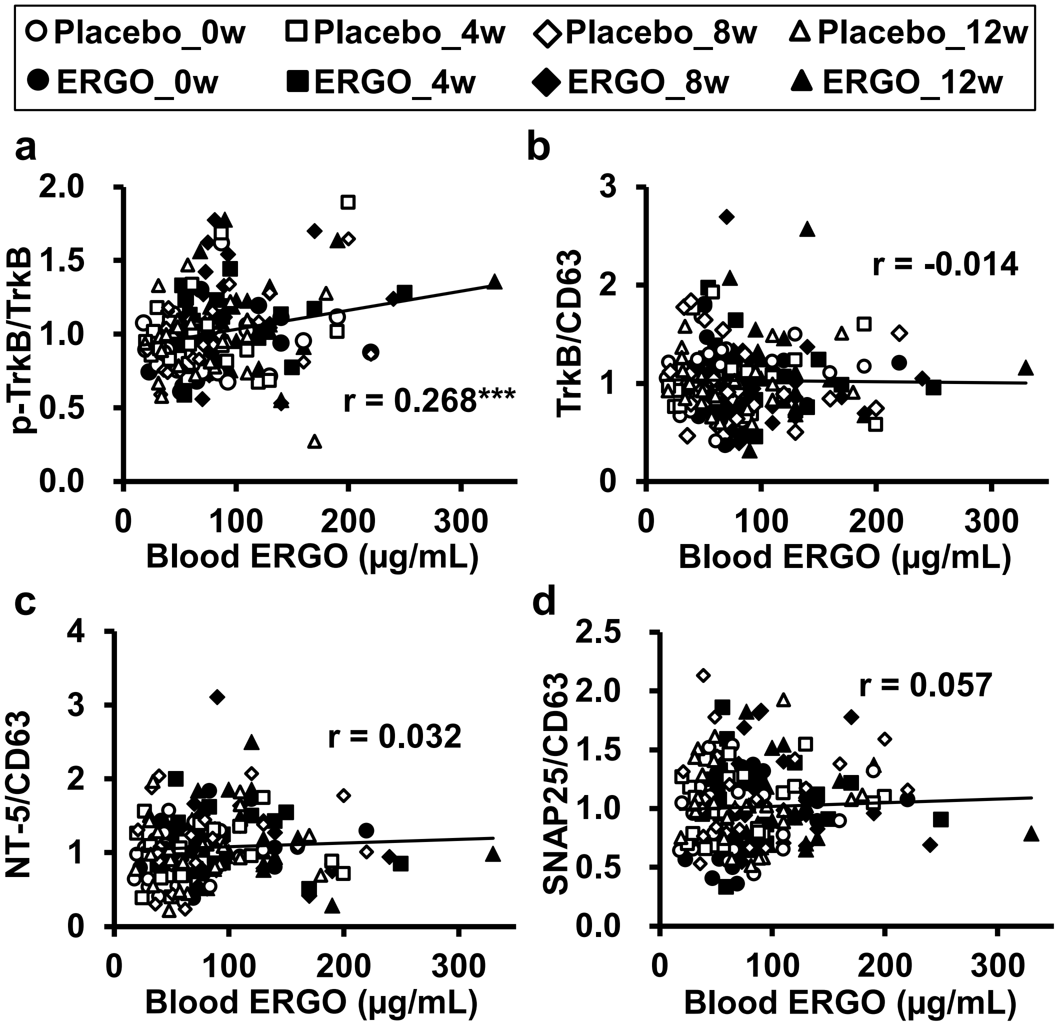

Supplemental Figure 5. Scatter plots showing the correlation between blood ERGO concentration and the ratio of each protein expression level in human serum EVs. The scatter plots include data for all samples obtained from the placebo and ERGO-treated groups at weeks 0, 4, 8, and 12 (placebo, n = 23; ERGO, n = 25). ***$P < 0.005$; correlations were determined using Pearson's correlation coefficient. r, correlation coefficient.

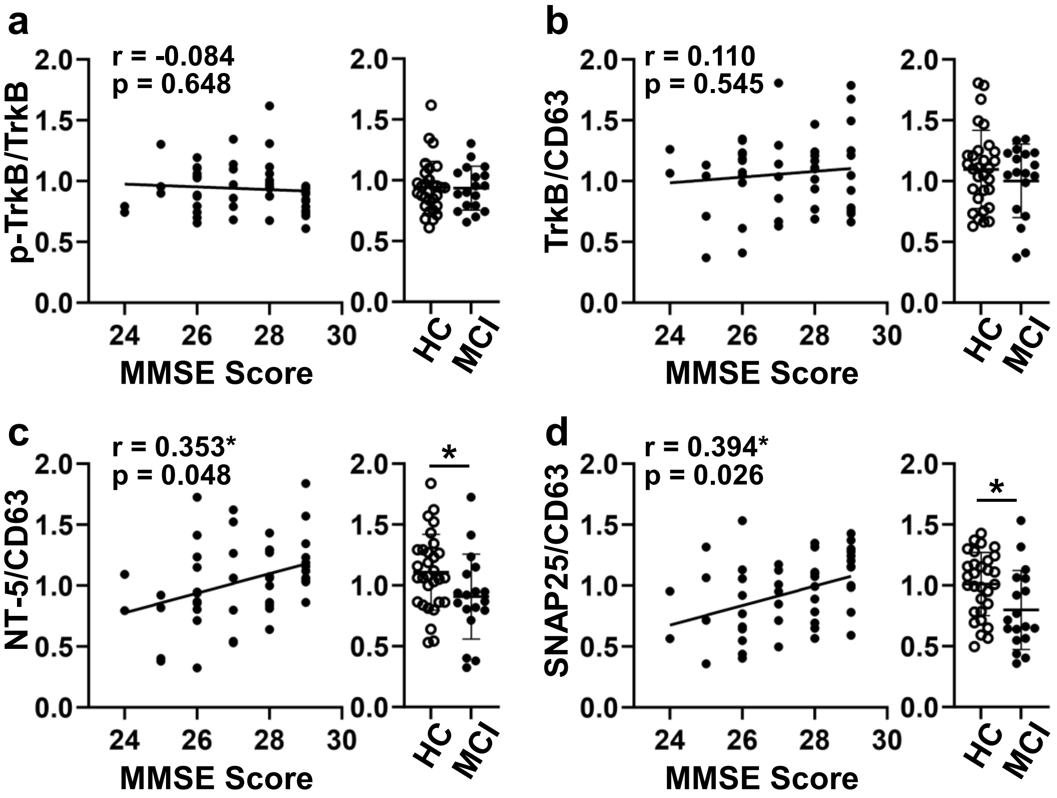

Supplemental Figure 6. Correlation between MMSE score and the ratio of each protein expression level in human serum EVs (p-TrkB/TrkB, TrkB/CD63, NT-5/CD63, and SNAP25/CD63), and difference in those ratios between healthy controls (HC) and participants with mild cognitive impairment (MCI). All data were obtained at week 0 before the start of the oral administration of the tablets. Correlations were determined using Pearson's correlation coefficient. r, correlation coefficient. Each value represents mean±standard deviation (SD, n = 30; MCI, n = 18). **P* < 0.05; Student's t-test.

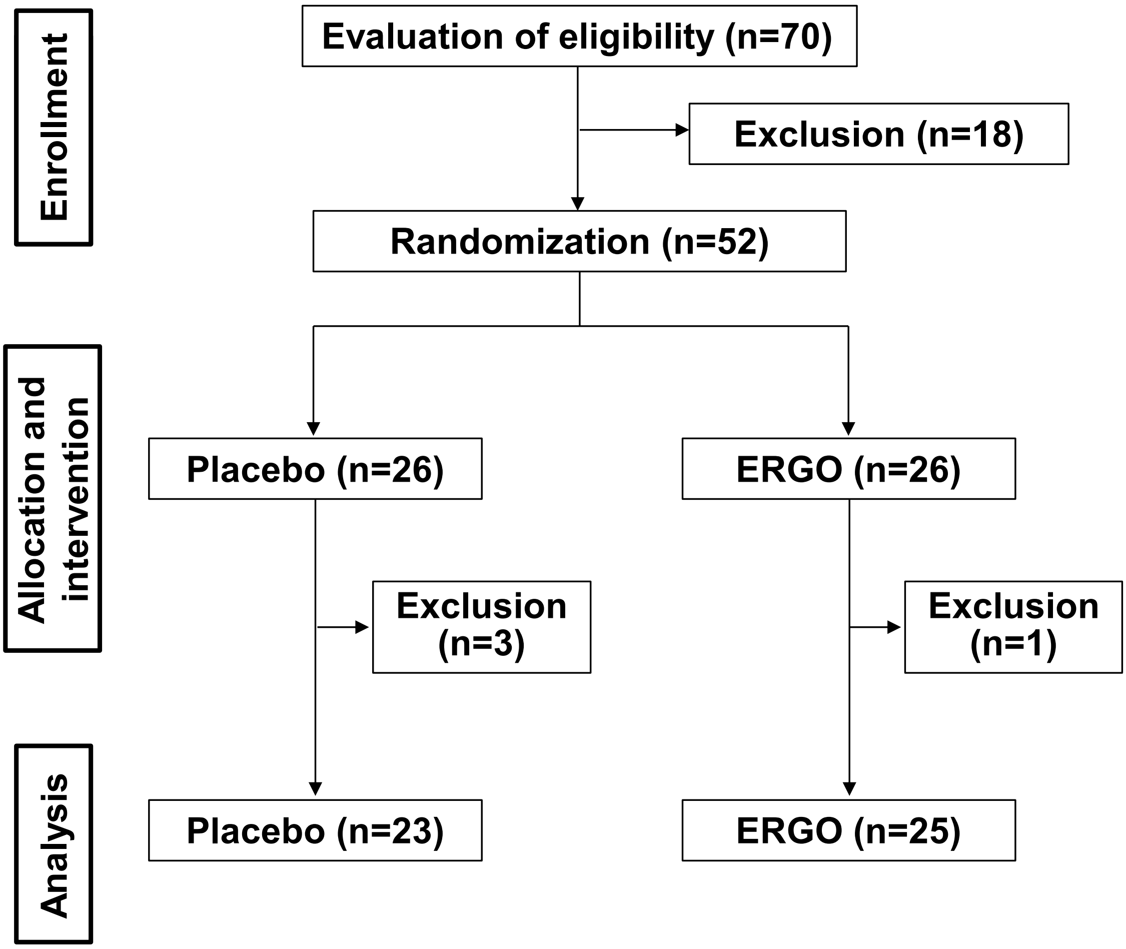

	Placebo (n=23)	ERGO (n=25)	*P*
**Male/Female**	5/18	11/14	0.102
**Age (years)**	44.2 ± 10.0	45.6 ± 9.3	0.631
**MMSE**	27.1 ± 1.2	27.3 ± 1.7	0.657

Supplemental Figure 7. Flow diagram of the criteria and procedure for selecting subjects during the trial. The exclusion of participants included those infected with the influenza virus (n = 2), who had missing data points (n = 1) and whose medication adherence was poor (n = 1).

Supplemental Table 1. The correlation coefficient between each cognitive domain score from Cognitrax and the ratio of each protein expression level in serum EVs or ERGO ΔAUC in all participants[1]

	p-TrkB /TrkB	TrkB /CD63	NT-5 /CD63	SNAP25 /CD63	ΔAUC for Serum ERGO[2]
Composite memory	0.279**	-0.080	-0.063	-0.078	0.224**
Verbal memory	0.230**	-0.084	-0.014	-0.033	0.199*
Processing speed	0.205**	-0.093	-0.068	-0.097	0.177*
Visual memory	0.210**	-0.042	-0.084	-0.089	0.173*
Psychomotor speed	0.140	-0.044	-0.117	-0.082	0.141
Reaction time	-0.129	-0.014	0.089	-0.029	-0.080
Complex attention	-0.164*	0.123	0.097	0.086	-0.112
Cognitive flexibility	0.199**	-0.082	-0.017	-0.066	0.087
Executive function	0.204**	-0.087	-0.013	-0.066	0.086
Working memory	0.187**	-0.054	-0.104	0.001	0.122

Sustained attention	0.089	-0.049	-0.137	-0.065	0.125
Simple attention	0.113	-0.152	-0.121	-0.061	0.115
Motor speed	0.035	0.016	-0.113	-0.042	0.062

[1]Correlations were determined using Pearson's correlation coefficient. [2]The area under the serum concentration curve (AUC) for ERGO was subtracted from that of the baseline obtained at week 0. **, $P < 0.01$; *, $P < 0.05$.

Supplemental Table 2. The correlation coefficients between each cognitive domain score from Cognitrax and the ratio of each protein expression level in serum EVs or ERGO ΔAUC in the placebo group[1]

	p-TrkB /TrkB	TrkB /CD63	NT-5 /CD63	SNAP25 /CD63	ΔAUC for Serum ERGO[1]
Composite memory	0.251*[2]	-0.118	-0.216*	-0.138	-0.013
Verbal memory	0.235*	-0.158	-0.185	-0.049	-0.080
Processing speed	0.156	-0.155	-0.153	-0.169	0.064
Visual memory	0.146	-0.024	-0.205	-0.082	-0.007
Psychomotor speed	0.022	-0.146	0.136	0.023	-0.246*
Reaction time	-0.069	0.080	0.183	0.074	-0.089
Complex attention	0.040	0.151	-0.044	-0.074	0.124
Cognitive flexibility	0.203	-0.078	-0.194	-0.165	0.060
Executive function	0.045	-0.090	-0.035	-0.078	0.135
Working memory	0.138	-0.101	-0.234*	0.014	0.038

Sustained attention	-0.014	-0.034	-0.174	-0.023	-0.029
Simple attention	0.100	-0.130	-0.208*	-0.070	0.088
Motor speed	0.051	-0.147	-0.156	-0.008	-0.047

[1]Correlations were determined using Pearson's correlation coefficient. [2]The area under the serum concentration curve (AUC) for ERGO was subtracted from that of the baseline obtained at week 0. *, $P < 0.05$.
