# Supplementary material for: TrkB phosphorylation in serum extracellular vesicles correlates with cognitive function enhanced by ergothioneine in humans
Source: NPJ Sci Food. 2024 Feb 6;8:11. doi: 10.1038/s41538-024-00250-5 (PMC10847428; doi:10.1038/s41538-024-00250-5)
Supplement: Supplementary file 2 — Reporting summary [file 41538_2024_250_MOESM2_ESM.pdf]

nature portfolio

Corresponding author(s): Yukio Kato

Last updated by author(s): Sep 29, 2023

Reporting Summary

Nature Portfolio wishes to improve the reproducibility of the work that we publish. This form provides structure for consistency and transparency in reporting. For further information on Nature Portfolio policies, see our Editorial Policies and the Editorial Policy Checklist.

Statistics

For all statistical analyses, confirm that the following items are present in the figure legend, table legend, main text, or Methods section.

n/a	Confirmed	
☐	☒	The exact sample size (*n*) for each experimental group/condition, given as a discrete number and unit of measurement
☐	☒	A statement on whether measurements were taken from distinct samples or whether the same sample was measured repeatedly
☐	☒	The statistical test(s) used AND whether they are one- or two-sided<br>*Only common tests should be described solely by name; describe more complex techniques in the Methods section.*
☐	☒	A description of all covariates tested
☐	☒	A description of any assumptions or corrections, such as tests of normality and adjustment for multiple comparisons
☐	☒	A full description of the statistical parameters including central tendency (e.g. means) or other basic estimates (e.g. regression coefficient) AND variation (e.g. standard deviation) or associated estimates of uncertainty (e.g. confidence intervals)
☐	☒	For null hypothesis testing, the test statistic (e.g. *F*, *t*, *r*) with confidence intervals, effect sizes, degrees of freedom and *P* value noted<br>*Give P values as exact values whenever suitable.*
☒	☐	For Bayesian analysis, information on the choice of priors and Markov chain Monte Carlo settings
☒	☐	For hierarchical and complex designs, identification of the appropriate level for tests and full reporting of outcomes
☐	☒	Estimates of effect sizes (e.g. Cohen's *d*, Pearson's *r*), indicating how they were calculated

Our web collection on statistics for biologists contains articles on many of the points above.

Software and code

Policy information about availability of computer code

Data collection	No software was used.
Data analysis	Data analysis were conducted using GraphPad Prism 7 (GraphPad Software, San Diego, CA, USA) and IBM-SPSS version 25. Figures were created by Graphpad Prism 7.

For manuscripts utilizing custom algorithms or software that are central to the research but not yet described in published literature, software must be made available to editors and reviewers. We strongly encourage code deposition in a community repository (e.g. GitHub). See the Nature Portfolio guidelines for submitting code & software for further information.

Data

Policy information about availability of data

All manuscripts must include a data availability statement. This statement should provide the following information, where applicable:

- Accession codes, unique identifiers, or web links for publicly available datasets
- A description of any restrictions on data availability
- For clinical datasets or third party data, please ensure that the statement adheres to our policy

All data generated or analyzed during this study are included in the article and its Supplementary Information.

Research involving human participants, their data, or biological material

Policy information about studies with human participants or human data. See also policy information about sex, gender (identity/presentation), and sexual orientation and race, ethnicity and racism.

Reporting on sex and gender	Male and Female
Reporting on race, ethnicity, or other socially relevant groupings	Japanese
Population characteristics	Age minimum: 20, Age maximum: 80
Recruitment	1) Females who are pregnant or lactating, and females who could become pregnant or lactating during test period<br>2) Subjects who are participating the other clinical tests. Subjects who participated within 3-month prior to the current study<br>3) Subjects who do not comply with instructions from the doctor or medical staff<br>4) Others who have been determined ineligible by investigator
Ethics oversight	Japan Food Evidence Association

Note that full information on the approval of the study protocol must also be provided in the manuscript.

Field-specific reporting

Please select the one below that is the best fit for your research. If you are not sure, read the appropriate sections before making your selection.

☒ Life sciences ☐ Behavioural & social sciences ☐ Ecological, evolutionary & environmental sciences

For a reference copy of the document with all sections, see nature.com/documents/nr-reporting-summary-flat.pdf

Life sciences study design

All studies must disclose on these points even when the disclosure is negative.

Sample size	The sample sizes were determined to be adequate based on the magnitude and consistency of measurable differences between groups. No statistical were used to predetermine sample sizes.
Data exclusions	No data were excluded from the analyses.
Replication	We confirm that all attempts at replication were successful.
Randomization	Samples were allocated at random using Microsoft Excel.
Blinding	Investigators were blinded to group allocation during data analysis.

Reporting for specific materials, systems and methods

We require information from authors about some types of materials, experimental systems and methods used in many studies. Here, indicate whether each material, system or method listed is relevant to your study. If you are not sure if a list item applies to your research, read the appropriate section before selecting a response.

Materials & experimental systems

n/a	Involved in the study	
☐	☒	Antibodies
☐	☒	Eukaryotic cell lines
☒	☐	Palaeontology and archaeology
☐	☒	Animals and other organisms
☒	☐	Clinical data
☒	☐	Dual use research of concern
☒	☐	Plants

Methods

n/a	Involved in the study	
☒	☐	ChIP-seq
☒	☐	Flow cytometry
☒	☐	MRI-based neuroimaging

Antibodies

Antibodies used	For immunostaining,

Antibodies used	1st antibodies: goat anti-doublecortin (Dcx) antibody (sc-8066, goat, Santa Cruz; Dallas, TX, USA) and mouse anti-NeuN antibody (MAB377, mouse, Merck Millipore; Burlington, MA)<br>2nd antibodies: anti-goat Alexa555 (A21432, donkey, Sigma-Aldrich; St. Louis, MO) and anti-mouse Alexa488 (A21202, donkey, Thermo Fisher Scientific; Waltham, MA)<br>For immunoprecipitation,<br>anti-SNAP25 antibody (sc-390644, mouse, Santa Cruz; Dallas, TX, USA)<br>For western blotting,<br>1st antibodies: anti-TrkB antibody (4603S, rabbit, Cell Signaling Technology; Danvers, MA), anti-p-TrkB antibody (ABN1381, rabbit, Merck Millipore; Burlington, MA), anti-NT-5 antibody (AB1781SP, rabbit, Merck Millipore; Burlington, MA), anti-SNAP25 antibody (sc-390644, mouse, Santa Cruz; Dallas, TX), anti-CD63 antibody (sc-15363, rabbit, Santa Cruz; Dallas, TX), anti-Flag antibody (14793S, rabbit, Cell Signaling Technology; Danvers, MA), and anti-β-actin antibody (A5441, mouse, Sigma-Aldrich; St. Louis, MO)<br>2nd antibodies: anti-rabbit IgG antibody conjugated with peroxidase (7074, goat, Cell Signaling Technology; Danvers, MA) or anti-mouse IgG antibody conjugated with peroxidase (A9917, goat, Sigma-Aldrich; St. Louis, MO)
Validation	sc-8066 (data-sheet and citations available), MAB377 (data-sheet and citations available), A21432 (data-sheet and citations available), A21202 (data-sheet and citations available), sc-390644 (data-sheet and citations available), 4603S (data-sheet and citations available), ABN1381 (data-sheet and citations available), AB1781SP (data-sheet and citations available), sc-15363 (data-sheet and citations available), 14793S (data-sheet and citations available), A5441 (data-sheet and citations available), 7074 (data-sheet and citations available), A9917 (data-sheet and citations available)

Eukaryotic cell lines

Policy information about cell lines and Sex and Gender in Research

Cell line source(s)	Neuro2a cells
Authentication	None of the cell lines used have been authenticated
Mycoplasma contamination	The Neuro2a cell line we used in this study was tested and found negative for mycoplasma contamination.
Commonly misidentified lines<br>(See ICLAC register)	No commonly misidentified cell line were used.

Animals and other research organisms

Policy information about studies involving animals; ARRIVE guidelines recommended for reporting animal research, and Sex and Gender in Research

Laboratory animals	Male ICR mice at 3-12 weeks of age
Wild animals	This study did not involve wild animals.
Reporting on sex	Male
Field-collected samples	This study did not involve filed collected samples.
Ethics oversight	All animal protocols were approved by the Committee on the Ethics of Animal Experiments of the University of Kanazawa (permit number: AP-183968) to minimize animal suffering and loss of life.

Note that full information on the approval of the study protocol must also be provided in the manuscript.
